# A Case of Reversible Posterior Leukoencephalopathy Syndrome (PRES) With a History of Migraine and Onset With Initial Visual Aura and Migraine-Like Headache, With a Significant Response to Lasmiditan: A Case Report

**DOI:** 10.7759/cureus.49311

**Published:** 2023-11-23

**Authors:** Yasutaka Sadamoto

**Affiliations:** 1 Neurosurgery, Headache Center, Takanoko Hospital, Matsuyama, JPN

**Keywords:** csd, endothelial cell, cortical spreading depolarization, pres, posterior reversible encephalopathy syndrome, visual aura, lasmiditan, migraine

## Abstract

Posterior reversible encephalopathy syndrome (PRES) is a neurological disease that presents with various neurological symptoms and is often accompanied by elevated blood pressure at onset. Neuroimaging, especially magnetic resonance imaging (MRI), often shows a characteristic parieto-occipital pattern with a symmetrical distribution of changes, reflecting vasogenic edema. Hypertension and endothelial cell damage are the most common causes of PRES. An association between migraine and endothelial cell damage has been suggested, but the relationship between migraine and PRES is unknown. Reports on PRES triggered by migraines are scarce.

We report a case of PRES in a 59-year-old woman with migraine without aura. At the onset, the patient experienced a first-ever visual aura and a migraine-like headache. In this case, it was also difficult to distinguish between PRES headache and headache caused by a pre-existing migraine; however, lasmiditan, an acute migraine treatment without vasoconstrictive properties, was remarkably effective for headaches.

## Introduction

Posterior reversible encephalopathy syndrome (PRES) is a neurological disease that presents with a variety of neurological symptoms including headaches, visual disturbances, seizures, and impaired consciousness and is often accompanied by elevated blood pressure at onset [1]. PRES is often reported as a complication of reversible cerebral vasoconstriction syndrome (RCVS) and is expected to have a common pathology [1,2]. PRES often presents with nonspecific symptoms; therefore, neuroimaging is necessary for diagnosis [1,2]. Magnetic resonance imaging (MRI) is more effective for computed tomography scanning because of its superior resolution, particularly for posterior fossa structures [1]. It often shows a characteristic parieto-occipital pattern with a symmetrical distribution of changes, reflecting vasogenic edema [2].

Hypertension and endothelial cell damage are the most common causes of PRES, and cytotoxic agents such as chemotherapy and immunosuppressive drugs, infections, (pre) eclampsia, and autoimmune diseases have been cited as causes of endothelial cell damage [2]. An association between migraine and endothelial cell damage has been suggested, especially in migraine with aura (MwA) [3]; however, the relationship between migraine and PRES is unknown. Reports about PRES occurring in migraine patients are scarce, as we researched.

We report a case of PRES in a 59-year-old woman with migraine without aura (MwoA). At the onset, the patient experienced a first-ever visual aura and a migraine-like headache. In this case, it was difficult to distinguish between PRES headache and headache caused by a pre-existing migraine; however, lasmiditan (REYBOW^®^), an acute migraine treatment without vasoconstrictive properties that activate the Serotonin/5-hydroxytryptamine (5-HT) 1F receptor [4], was remarkably effective.

## Case presentation

A 59-year-old woman with a body mass index of 20.94 (kg/m^2^). The patient had a habit of drinking 350 ml of beer approximately three days a week and did not smoke. The patient had a history of vertigo attacks around the age of 30 and was diagnosed with Meniere's disease by a local otolaryngologist. On September 1, 2020, the patient experienced a vertigo attack for a few tens of seconds upon waking up; however, this did not recur. The patient was not using any drugs regularly. The patient underwent regular annual physical examinations. In June 2022, her blood pressure was 100/67 mmHg, and no abnormal findings were noted on the electrocardiogram or plain chest scan; blood tests showed that her liver and kidney function parameters and blood glucose levels were within normal ranges. The patient did not report dyslipidemia or any specific symptoms of anxiety or depression.

The patient had experienced headache attacks once or twice a month since she was approximately 22 years old. Headache tended to occur on busy days or holidays, appeared bilaterally temporal to the occipital areas, and was pulsatile. Headache attacks are aggravated by daily activities and accompanied by nausea, photophobia, phonophobia, and osmophobia. However, the headache was not accompanied by any aura, such as visual, motor, sensory, or speech, and was not accompanied by any facial autonomic symptoms such as lacrimation, nasal discharge, or nasal obstruction. On a numerical rating scale of 1-10, the pain had an intensity of 6/10. Without the use of acute-care medications, headaches lasted from half a day to a day, prevented her from doing her chores, and the patient went to bed for a day. Although her headache sometimes interfered with her daily life, the patient did not visit a headache specialist or consult her internist because her headache was relieved within two hours after taking over-the-counter aspirin. Therefore, the patient was not diagnosed with migraine and had never taken triptans internally.

On May 21, 2023, the patient was cleaning up after dinner, and her visual field in the center darkened slowly, gradually extending peripherally, with a zigzagging scintillated edge. This was symmetrical in both eyes. Her visual field abnormalities gradually disappeared after approximately 15 minutes. Subsequently, the patient developed a pulsating headache of 6/10 on a numerical rating scale from the bilateral temporal to bilateral occipital areas with nausea. The patient took 500mg of aspirin and went to bed with a 4-5/10 headache after two hours. However, the patient continued to have a 5-6/10 headache after waking the next morning and visited a headache specialist at our hospital.

**Figure 1:**
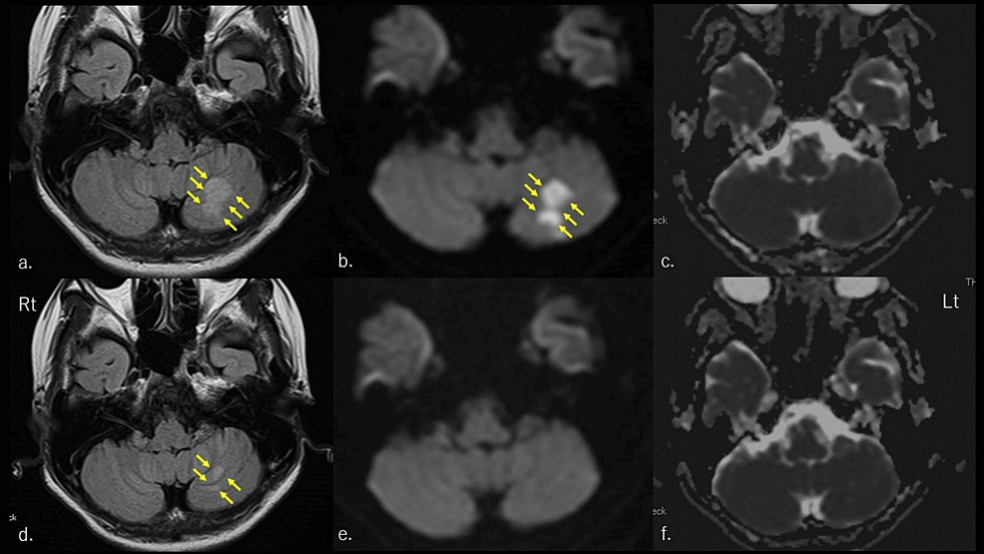
Head magnetic resonance imaging. The upper-row images are taken during the initial presentation. Arrows point to hyperintensities associated with PRES. (a) FLAIR sequences showing hyperintensities in the left cerebellar regions. (b) DWI showing hyperintensities in the left cerebellar regions. We considered it is T2 shine through. (c) ADC maps show a normal appearance in the left cerebellar regions. The lower-row images were taken in the third week after disease onset. (d) FLAIR sequences showing slight hyperintensities in the left cerebellar regions. (e, f) DWI and ADC maps showing normal appearance in the left cerebellar regions. PRES: posterior reversible encephalopathy syndrome; FLAIR: fluid-attenuated inversion recovery; DWI: diffusion-weighted imaging, ADC: apparent diffusion coefficient

When the patient visited our department, her level of consciousness was clear and her blood pressure was 156/97 mmHg, which was higher than her usual blood pressure. Her temperature was 36.6 °C and her pulse rate was 97/min. No abnormalities were observed in the pupils, deep tendon reflexes, or other neurological features. The patient experienced the first visual aura and continued to have headaches at the time of the visit; therefore, a brain MRI was performed. MRI revealed hyperintensities in the inferior part of the left cerebellar lobule on fluid-attenuated inversion recovery (FLAIR) sequences and diffusion-weighted images (DWI) (Figure 1a, 1b). However, apparent diffusion coefficient (ADC) maps did not show any signal reduction in the same area (Figure 1c). We considered the hyperintensities observed on DWI to be T2 shine-through [5] and suspected PRES. From the history interview, we diagnosed her previous headache attacks as MwoA, based on the International Classification of Headache Disorders, Third Edition (2018) [6]. Her headache was similar in nature to her previous recurrent headache attacks, except for visual aura, long duration, and poor response to aspirin. Her headache persisted at the time of examination, and we considered it a symptom of PRES. However, it was also possible that her pre-existing migraine was exacerbated by PRES. Therefore, we considered the use of triptans or lasmiditan for acute treatment of migraine. In Japan, insurance coverage for triptans is contraindicated for ischemic heart diseases, such as myocardial infarction and cerebrovascular disease, including transient ischemic attacks. Dizziness, somnolence, and malaise have been cited as side effects of lasmiditan. The patient had a history of vertigo. However, the patient had not experienced a recurrence of vertigo since her last vertigo attack on September 1, 2020. Based on the initial brain MRI findings, we had to consider the possibility of cerebral infarction as well as PRES. We explained the possibility of recurrence of vertigo to the patient and obtained her consent for a lasmiditan prescription. Finally, we chose lasmiditan instead of triptan and prescribed 50 mg of lasmiditan. Her headache disappeared 30 minutes after drug administration. No adverse events, such as dizziness, vertigo, somnolence, and malaise occurred. Verapamil hydrochloride (240 mg/day) was prescribed because of the risk of RCVS complications in PRES and the prevention of preexisting migraine. The patient experienced her usual (without aura) headache attacks on May 22 at night and June 27, both of which were relieved within 30 minutes with oral 100mg of lasmiditan without any adverse events. We retested the brain MRI three weeks after the onset of aura and found a reduction in the hyperintensities on FLAIR sequences and the disappearance of the hyperintensities on DWI (Figure 1d, 1e). No obvious cerebral artery stenosis was observed on the first or second head magnetic resonance angiography (MRA) (data not shown). We made the final diagnosis of PRES associated with MwoA. Because of the lack of apparent RCVS and the frequency of migraine attacks once or twice a month, verapamil hydrochloride was withdrawn from the first month of onset.

## Discussion

PRES develops most frequently in young or middle-aged adults, with a mean age of 45 years, and appears to be predominantly female [1]. This phenomenon was first described by Hinchey et al. in 1996 [2]. Hypertension and endothelial cell damage are the most common causes of PRES [2]. PRES presents with various clinical manifestations and is primarily characterized by vasogenic edema of the parietal and occipital regions on imaging [7]. The posterior circulation may be more vulnerable to hyperperfusion because it has less sympathetic innervation than counterreflex parasympathetic vasodilation [1]. The differential diagnosis of PRES is wide-ranging and includes conditions with similar confluent T2-weighted images (T2WI) or FLAIR sequences white matter hyperintensities. Examples include infarction, demyelinating diseases, infections, progressive multifocal leukoencephalopathy, vasculitis, and various metabolic disorders [8]. In the present case, brain MRI revealed hyperintensities on FLAIR sequences and DWI in the left cerebellum and iso intensities on ADC maps in the same area (Figure 1a, 1b, 1c). The incidence of regions of involvement was parietooccipital (98.7%), posterior frontal (78.9%), temporal (68.4%), thalamus (30.3%), cerebellum (34.2%), brainstem (18.4%) and basal ganglia (11.8%) [5]. Another study that examined the atypical site of onset in PRES is found to be more common in the cerebellum [9]. Furthermore, unilateral PRES is rare [10]. Vasogenic edema, which is an essential pathological feature of PRES, is usually hypointense on T1-weighted images, hyperintense on T2WI and FLAIR sequences, and isointense or hyperintense on DWI and ADC maps. Hyperintensity on DWI and hypointensity on ADC maps, which is called restricted diffusion, can reflect cytotoxic edema [9]. We considered the hyperintensities observed on DWI to be T2 shine-through [5]. Brain MRI at the third week of onset showed a decrease in the hyperintensities of the FLAIR sequences and fading of the hyperintensities on DWI. ADC maps showed iso intensities, as in the first case. We diagnosed PRES based on these changes in MRI findings.

Migraine is a highly disabling and common neurological disorder characterized by a complex neurobiology involving a series of regions and networks in the central and peripheral nervous systems [11]. Accumulating evidence implicates endothelial activation mediated by oxidative stress in female migraineurs [12], which has also been implicated in endothelial cell damage [3]. Migraine is associated with inflammation and a procoagulant environment, cerebrovascular disorders have been suggested to be associated with cerebral infarction, RCVS, and carotid artery dissection [13]. Several studies have reported that migraineurs exhibit functional abnormalities in the cerebellum, migraine research also found an increased prevalence of ischemic lesions particularly in the cerebellar posterior lobe of migraineurs [14]. Furthermore, the cerebellum exhibited remarkably high concentrations of calcitonin gene-related peptide (CGRP) [14].

In the present case, at the onset, the patient experienced a first-ever visual aura and a migraine-like headache. Patients with PRES often report blurred vision, hemianopia, visual neglect, and frank cortical blindness [15]. Many of the reports in PRES are long-lasting binocular visual disorders [16]. The visual disturbance in this case was typical of migraine visual aura-like symptoms. Migraine aura-like symptoms have also been reported in ischemic stroke [17,18]. In patients with aura, the need to differentiate between the following diseases is mentioned: ischemic lesions, inflammatory diseases, migrainous infarction, epileptic disorders, neoplastic lesions, amyloid angiopathy, subdural hematoma, RCVS, and PRES [6,16]. However, the relationship between PRES and visual aura is unknown. Cortical spreading depolarization (CSD), the pathophysiological and electrophysiological basis of migraine auras, has also been observed in patients with acute ischemic stroke [18]. CSD is hypothesized to directly trigger headaches by activating trigeminal nociceptive fibers in the dura mater through the release of noxious substances including potassium and nitric oxide [19]. In the present case, we hypothesized that PRES may be associated with migraine, based on possible endothelial cell damage. Although the mechanism is unknown, it is thought that CSD was induced during the onset of PRES, resulting in a visual aura. It was difficult to determine whether the migraine-like headache was due to PRES or the exacerbation of a preexisting migraine. Since the prevalence of migraine is high (approximately 10 %) [20], PRES may occur incidentally in migraine patients, and further case studies are needed to examine the association between migraine, aura, and PRES.

Treatment of PRES is typically aimed at controlling the primary etiology causing PRES [21]. In this case, treatment for migraine was considered necessary. Headache of migraine begins peripherally when nociceptive neurons that innervate the dura mater are stimulated and release vasoactive neuropeptides such as CGRP and pituitary adenylate cyclase-activating polypeptide-38, causing signaling along the trigeminovascular pathway [20]. Triptans, which are mainly used for the acute treatment of migraines, activate 5-HT mainly 1D, and 1F receptors in the periphery, and inhibit the presynaptic release of CGRP from trigeminal nerve endings [22]. However, triptans are associated with the risk of coronary artery constriction because of their effects on the 5-HT 1B receptor [23]. The 5-HT 1B receptors are present in the smooth muscle and endothelium of the human middle dural and cerebral arteries [23]. RCVS, which has been implicated in the pathogenesis of PRES [1], is associated with the use of triptans [23]. Furthermore, in PRES it has been noted that vasoconstriction may occur in response to edema [5].

Lasmiditan exhibits high selectivity for 5-HT 1F receptors [4,24]. 5-HT 1F receptors are present in the meninges, trigeminal ganglia, trigeminal nucleus caudalis, hypothalamus, thalamus, cortex, and other central nerves involved in the regulation of migraine pain signaling, as well as in peripheral trigeminal nerve endings [4]. Lasmiditan activates 5-HT 1F receptors in the periphery and inhibits the presynaptic release of CGRP from trigeminal nerve endings [4]. It acts on 5-HT 1F receptors in the central nervous system through the blood-brain barrier, modulating central sensitization in the trigeminal nucleus caudalis and thalamus [4]. Moreover, lasmiditan does not affect 5-HT 1B receptor [4]. Therefore, lasmiditan has been suggested to be highly effective in managing migraines in patients with vascular disorders. Adverse events such as dizziness, somnolence, and malaise have been reported with lasmiditan, possibly due to its central effects [4]. As in this case, in migraine patients with new or potential cerebrovascular disease who have headaches at presentation, it is important to improve headaches because of the need to control sympathetic overexcitation and maintain restfulness during brain MRI. In this regard, we believe that the benefits of using lasmiditan after explaining its possible adverse events to the patient outweigh the possible adverse events.

## Conclusions

Migraine, especially MwA in women, may be associated with cerebrovascular disease. This is assumed to be related to PRES. When an unusual headache occurs in a patient with a history of migraine, organic diseases should be ruled out. It can be difficult to determine whether a new headache is due to an organic disease or a history of migraine. Lasmiditan is an effective option for acute treatment of migraines with or assumed to be associated with cerebrovascular disease when aspirin, paracetamol, and nonsteroidal anti-inflammatory drugs are ineffective.

## References

[1] Triplett JD, Kutlubaev MA, Kermode AG, Hardy T (2022). Posterior reversible encephalopathy syndrome (PRES): diagnosis and management. Pract Neurol.

[2] Anderson RC, Patel V, Sheikh-Bahaei N (2020). Posterior Reversible Encephalopathy Syndrome (PRES): pathophysiology and neuro-imaging. Front Neurol.

[3] Paolucci M, Altamura C, Vernieri F (2021). The role of endothelial dysfunction in the pathophysiology and cerebrovascular effects of migraine: a narrative review. J Clin Neurol.

[4] Clemow DB, Johnson KW, Hochstetler HM, Ossipov MH, Hake AM, Blumenfeld AM (2020). Lasmiditan mechanism of action - review of a selective 5-HT(1F) agonist. J Headache Pain.

[5] McKinney AM, Short J, Truwit CL, McKinney ZJ, Kozak OS, SantaCruz KS, Teksam M (2007). Posterior reversible encephalopathy syndrome: incidence of atypical regions of involvement and imaging findings. AJR Am J Roentgenol.

[6] (2018). Headache Classification Committee of the International Headache Society (IHS) The International Classification of Headache Disorders, 3rd edition. Cephalalgia.

[7] Fischer M, Schmutzhard E (2017). Posterior reversible encephalopathy syndrome. J Neurol.

[8] Hugonnet E, Da Ines D, Boby H, Claise B, Petitcolin V, Lannareix V, Garcier JM (2013). Posterior reversible encephalopathy syndrome (PRES): features on CT and MR imaging. Diagn Interv Imaging.

[9] Li K, Yang Y, Guo D, Sun D, Li C (2020). Clinical and MRI features of posterior reversible encephalopathy syndrome with atypical regions: a descriptive study with a large sample size. Front Neurol.

[10] Ozawa T, Tanaka R, Nagaoka R (2019). Data on characteristics of reported cases of unilateral posterior reversible encephalopathy syndrome (PRES). Data Brief.

[11] Puledda F, Silva EM, Suwanlaong K, Goadsby PJ (2023). Migraine: from pathophysiology to treatment. J Neurol.

[12] Tietjen GE, Maly EF (2020). Migraine and ischemic stroke in women. A narrative review. Headache.

[13] Mawet J, Debette S, Bousser MG, Ducros A (2016). The link between migraine, reversible cerebral vasoconstriction syndrome and cervical artery dissection. Headache.

[14] Qin Z, He XW, Zhang J (2019). Structural changes of cerebellum and brainstem in migraine without aura. J Headache Pain.

[15] Hinchey J, Chaves C, Appignani B (1996). A reversible posterior leukoencephalopathy syndrome. N Engl J Med.

[16] Eren OE, Wilhelm H, Schankin CJ, Straube A (2021). Visual phenomena associated with migraine and their differential diagnosis. Dtsch Arztebl Int.

[17] Waters MJ, Cheong E, Jannes J, Kleinig T (2018). Ischaemic stroke may symptomatically manifest as migraine aura. J Clin Neurosci.

[18] Scutelnic A, Kreis LA, Beyeler M (2023). Migraine aura-like symptoms at onset of stroke and stroke-like symptoms in migraine with aura. Front Neurol.

[19] Thomsen AV, Puonti O, Gaist D (2023). Investigations of the subarachnoid space as a potential link between aura and headache in migraine: a case-control MRI study. Cephalalgia.

[20] Dodick DW (2018). A phase-by-phase review of migraine pathophysiology. Headache.

[21] Granata G, Greco A, Iannella G, Granata M, Manno A, Savastano E, Magliulo G (2015). Posterior reversible encephalopathy syndrome--Insight into pathogenesis, clinical variants and treatment approaches. Autoimmun Rev.

[22] Rubio-Beltrán E, Labastida-Ramírez A, Villalón CM, MaassenVanDenBrink A (2018). Is selective 5-HT(1F) receptor agonism an entity apart from that of the triptans in antimigraine therapy?. Pharmacol Ther.

[23] Leroux E, Rothrock J (2019). Triptans for migraine patients with vascular risks: new insights, new options. Headache.

[24] Nelson DL, Phebus LA, Johnson KW, Wainscott DB, Cohen ML, Calligaro DO, Xu YC (2010). Preclinical pharmacological profile of the selective 5-HT1F receptor agonist lasmiditan. Cephalalgia.

